# Autosomal Dominant Hypocalcemia (Hypoparathyroidism) Types 1 and 2

**DOI:** 10.3389/fphys.2016.00458

**Published:** 2016-10-18

**Authors:** Kelly L. Roszko, Ruiye D. Bi, Michael Mannstadt

**Affiliations:** Endocrine Unit, Massachusetts General Hospital and Harvard Medical SchoolBoston, MA, USA

**Keywords:** autosomal-dominant hypocalcemia, CASR, calcium metabolism, GNA11, G11, calcilytics, YM254890, hypocalcemia

## Abstract

Extracellular calcium is essential for life and its concentration in the blood is maintained within a narrow range. This is achieved by a feedback loop that receives input from the calcium-sensing receptor (CASR), expressed on the surface of parathyroid cells. In response to low ionized calcium, the parathyroids increase secretion of parathyroid hormone (PTH) which increases serum calcium. The CASR is also highly expressed in the kidneys, where it regulates the reabsorption of calcium from the primary filtrate. Autosomal dominant hypocalcemia (ADH) type 1 is caused by heterozygous activating mutations in the CASR which increase the sensitivity of the CASR to extracellular ionized calcium. Consequently, PTH synthesis and secretion are suppressed at normal ionized calcium concentrations. Patients present with hypocalcemia, hyperphosphatemia, low magnesium levels, and low or low-normal levels of PTH. Urinary calcium excretion is typically increased due to the decrease in circulating PTH concentrations and by the activation of the renal tubular CASR. Therapeutic attempts using CASR antagonists (calcilytics) to treat ADH are currently under investigation. Recently, heterozygous mutations in the alpha subunit of the G protein G11 (Gα11) have been identified in patients with ADH, and this has been classified as ADH type 2. ADH2 mutations lead to a gain-of-function of Gα11, a key mediator of CASR signaling. Therefore, the mechanism of hypocalcemia appears similar to that of activating mutations in the CASR, namely an increase in the sensitivity of parathyroid cells to extracellular ionized calcium. Studies of activating mutations in the CASR and gain-of-function mutations in Gα11 can help define new drug targets and improve medical management of patients with ADH types 1 and 2.

## Physiology of extracellular calcium homeostasis

Calcium is vital for many functions of the body and blood calcium concentrations must be maintained within a narrow physiological range of 8.5–10.5 mg/dl (2.12–2.62 mmol/L). The calcium-sensing receptor (CASR), a class C G-protein coupled receptor comprised of 1078 amino acids, is an integral component of the homeostatic system that controls blood calcium concentrations (Hofer and Brown, 2003). In humans, the two major calcium-controlling hormones that maintain serum calcium within the normal range are parathyroid hormone (PTH) and 1,25-dihydroxyvitamin D. PTH is produced and secreted by the parathyroid glands, which sense extracellular calcium through the calcium sensing receptor, located on the surface of the parathyroid chief cells (Brown et al., 1993).

When serum ionized calcium is low, increased amounts of PTH are secreted into the circulation; PTH acts mainly on kidney and bone, through binding and activating the PTH/PTHrP receptor, to increase serum ionized calcium. In bone, PTH induces osteoblasts to trigger osteoclasts to release calcium and phosphorous into the circulation, and it stimulates osteocytic osteolysis (Tazawa et al., 2004). In addition, PTH acts at the distal tubules of the kidneys to induce calcium reabsorption. In the proximal tubule, PTH inhibits phosphate reabsorption from the urine resulting in a decrease in serum phosphate levels. PTH also activates the 1α-hydroxylase enzyme (CYP27B1) in the renal proximal tubule, which converts 25-hydroxyvitamin D to the active form, 1,25-dihydroxyvitamin D, or calcitriol. This active vitamin D functions in the intestine to increase the absorption of calcium (and phosphate) from the gut. Taken together, these concerted actions of PTH mobilize calcium in an effort to restore the serum calcium to the above-mentioned normal range.

## CASR and parathyroid cells

The calcium sensing receptor is expressed in most tissues (Brown et al., 1993). However, the highest levels of its expression are found in the parathyroid glands and in the kidneys. The minute-to-minute tight regulation of calcium is achieved through the ability of the CASR to sense minor changes in extracellular calcium. The relationship between PTH secretion and the extracellular calcium concentration has been described as a steep sigmoidal curve. In the parathyroid cell, activation of the CASR by extracellular ionized calcium stimulates intracellular signal transduction pathways, resulting in an increase in intracellular calcium (nM range), that ultimately leads to an inhibition of PTH production and secretion. This is in stark contrast to the usual signaling pattern of intracellular calcium, which is characterized by the stimulation of hormone production and secretion in response to an increase in intracellular calcium. The mechanism for this peculiar opposite effect in the parathyroids is unknown.

Several signaling pathways downstream of the CASR are engaged in the parathyroid cell. In the presence of ligand, the CASR activates Gq and G11(Hofer and Brown, 2003), leading to a stimulation of phospholipase C (PLC). PLC hydrolyzes phosphatidylinositol 4,5-bisphosphate (PIP2) to diacyl glycerol and inositol 1,4,5-trisphosphate (IP3), which in turn causes activation of protein kinase C (PKC) and release of intracellular calcium, respectively. The CASR also couples to the G-protein Gi, which inhibits adenylate cyclase and leads to a reduction in intracellular cAMP.

In addition to extracellular calcium, the parathyroids also respond to phosphate, 1,25-dihydroxyvitamin D, and FGF23, both directly and indirectly, to modulate PTH secretion (Naveh-Many et al., 2002; Silver and Naveh-Many, 2009).

## CASR and the kidneys

In the kidney, the CASR is highly expressed in the basolateral membrane of the thick ascending limb (TAL; Loupy et al., 2012; Toka et al., 2012; Crisi et al., 2013). While there is conflicting evidence for the expression of CASR mRNA and protein in other parts of the nephron, functional studies suggest that it is more widely expressed. For example, in the collecting duct, CASR activation leads to urine acidification and a reduction in water reabsorption, which could reduce the risk of kidney stone formation (Renkema et al., 2009).

In the TAL, calcium is reabsorbed passively along the paracellular pathway, driven by luminal electropositivity, which is dependent on the rate and extent of NaCl reabsorption. In the apical membrane of the TAL, Na^+^, 2Cl^−^, and K^+^ are electroneutrally transported into the cell. K^+^ is recycled back into the lumen through the apical ROMK channel leading to lumen positivity, the main driver of paracellular calcium transport from the lumen into the TAL. In the distal convoluted tubule (DCT), calcium is reabsorbed through an active transcellular transport. Calcium enters the DCT through TRPV5 and exits the cell via the sodium-calcium exchanger 1 (Riccardi and Valenti, 2016).

Activation of the CASR in the kidney leads to a decrease in calcium (and water and sodium) reabsorption. While these exact pathways are not entirely elucidated, two potential mechanisms have emerged. One involves the paracellular transport of calcium from the lumen into the TAL, which is dependent on several claudins, a family of proteins that establish barriers in tight junctions. The CASR was discovered to control claudin-14 expression and therefore absorption of calcium through the paracellular tight junctions (Gong and Hou, 2014). The second possible mechanism is a reduction of the activity of the apical K^+^ channel, which generates the lumen positivity. Patch clamp studies showed that both calcium and neomycin are able to reduce the activity of this potassium channel (Wang et al., 1996, 1997).

Investigators studying CASR knockout mouse models demonstrated that the CASR also defends against hypercalcemia by increasing calcium excretion by the kidney, independent of its role in the parathyroid glands (Kantham et al., 2009).

## Autosomal-dominant hypocalcemia type 1

Key to the understanding of the role of the CASR in the parathyroids was the discovery of mutations in the *CASR* gene leading to human disease, and analysis of these mutations in mouse models (Hannan and Thakker, 2013). Patients with activating or inactivating germline mutations in the CASR present with hypocalcemia or hypercalcemia, respectively. Inactivating mutations of the CASR lead to familial hypocalciuric hypercalcemia (FHH). The mirror image of FHH, autosomal-dominant hypocalcemia (ADH) type 1, is caused by activating mutations in the CASR and is the most common genetic form of isolated hypoparathyroidism. These activating CASR mutations lead to a leftward shift in the calcium-PTH curve and therefore suppression of PTH secretion at physiological levels of extracellular calcium. Biochemical hallmarks of AHD1 are hypocalcemia, which is typically mild to moderate, hyperphosphatemia, hypercalciuria, and inappropriately low but detectable PTH levels. Symptoms of ADH1 are caused by hypocalcemia (mainly neuromuscular irritability) and are typically mild.

In addition to this functional defect in the parathyroids, activating CASR mutations have independent effects in the kidneys. Therefore, patients with ADH1 have two mechanisms contributing to hypercalciuria. First, low concentrations of PTH, which normally induce reabsorption of calcium from the primary filtrate, result in relative hypercalciuria. Second, increased activation of the mutated CASR through extracellular calcium in the distal renal tubules leads to even more pronounced hypercalciuria for any given blood calcium level.

The presentation of the index case of kindred G (D'Souza-Li et al., 2002) is typical for ADH1. Blood chemistries of this 21-year old asymptomatic woman were tested because her three sisters and her mother all had hypocalcemia. Her laboratory results showed mild hypocalcemia (Ca = 7.5 mg/dl, normal 8.5–10.5), mild hyperphosphatemia (P = 4.8 mg/dl, normal 2.6–4.5) and hypomagnesemia (Mg = 1.4 mg/dl, normal 1.8–2.5), low but detectable PTH (PTH = 16 pg/ml, normal 10–60) and an elevated calcium/creatinine clearance ratio (0.088, normal < 0.02). Sanger sequencing revealed a heterozygous missense mutation leading to the substitution of alanine to threonine in position 835, located in the third extracellular loop of the CASR. *In vitro* studies using HEK cells transfected with wildtype and mutant CASR cDNA revealed the expected leftward shift in the calcium-response curve (D'Souza-Li et al., 2002).

Diagnostic sequencing of the CASR gene is used to confirm ADH1. More than 200 mutations of the CASR have been reported, of which more than 70 are associated with ADH1, the vast majority are heterozygous missense mutations (www.casrdb.mcgill.ca). The CASR consists of three major domains: the large extracellular domain (ECD), a transmembrane domain (TMD), and an intracellular C-terminus. Many mutations associated with ADH1 are located in the second peptide loop of the ECD, which is predicted to be important for dimer formation, as well as in the TMD 5 and 6 and in the region of the third extracellular loop.

Clinical management of ADH1 is guided by the known high risk for renal calcifications, kidney stones and kidney failure. In asymptomatic patients, treatment should be avoided. When hypocalcemic symptoms occur frequently enough to warrant treatment, careful therapy with the lowest amount of calcium and activated vitamin D is initiated. Goal calcium levels should be as low as possible to alleviate symptoms. Thiazide diuretics, often used in hypoparathyroidism because of their urinary calcium lowering effect, have also been shown to be beneficial in ADH1 (Sato et al., 2002). In clinical studies in patients with ADH1, once daily dosing of PTH(1–34) only corrected serum calcium for a part of the day, and twice daily sc administration of PTH(1-34) led to better control of blood calcium (Winer et al., 1998, 2008). However, PTH does not correct the effects of the activated CASR in the kidneys, and indeed, urinary calcium excretion was not normalized by PTH(1–34) injections. Studies of continuous PTH(1–34) administration via pump therapy did normalize urinary calcium in cases of postsurgical hypoparathyroidism (Winer et al., 2012). Trials with PTH(1–84), which is an approved treatment for other forms of hypoparathyroidism, excluded patients with ADH1.

Small molecule allosteric modulators of the CASR have been identified (Nemeth and Goodman, 2016). Positive modulators (type II calcimimetics), such as cinacalcet, increase activation of the CASR. Cinacalcet is primarily used for treatment of patients with secondary hyperparathyroidism on dialysis. Calcilytics, which are negative allosteric CASR modulators, inhibit the activation of the CASR and therefore are of potential interest for the treatment of ADH1. In cell culture experiments studying activating CASR mutants, calcilytics have been shown to normalize the leftward shift of the calcium response curve (Dong et al., 2015; Hannan et al., 2015). The utility of calcilytics was further demonstrated in two studies in mice harboring activating CASR mutations. In one study, two knockin mouse models of ADH1 with activating mutations in the CASR were generated. Daily oral administration of the calcilytic JTT-305/MK-5442 to these mice was shown to increase PTH and calcium, and to reduce urinary calcium excretion (Dong et al., 2015). A second study using the Nuf mouse, another model of ADH1, showed that intraperitoneal injection of the calcilytic NPS2143 transiently stimulated serum PTH secretion and increased serum calcium without increasing urinary calcium (Hannan et al., 2015). In a phase 2 clinical trial, IV administration of the structurally closely related small molecule NPSP795 increased plasma PTH levels and decreased the urinary fractional excretion of calcium in five patients with ADH1 (Ramnitz et al., 2015). In addition to a potential treatment for ADH1, calcilytics could also have a role in reducing urinary excretion of calcium in other patients with hypoparathyroidism, through blocking the renal CASR.

## Autosomal-dominant hypocalcemia type 2

With the discovery that mutations in the gene encoding the alpha subunit of the G protein G11 (*GNA11*) cause hypocalcemia, ADH was divided into type 1, reviewed above, and type 2, caused by activating mutations in *GNA11*. Patients with ADH2 present with a wide range of hypocalcemic symptoms ranging from paresthesias to tetany and seizures, or they can be asymptomatic.

The following describes the index case of Family A (Mannstadt et al., 2013): A 15 year-old boy presented with muscle cramps and tremulousness. Past laboratory studies revealed normal calcium at age 2. He experienced generalized seizures at age 5 which were treated with carbamazepine for 1 year and did not re-occur thereafter. Studies on presentation revealed mildly low serum calcium, high phosphorous, and inappropriately low PTH concentration (Table 1).

**Table 1:** **Summary of the presenting laboratory values of the index case of Family A, a 15 year-old boy with ADH2, from Mannstadt et al. (2013)**.

	**At presentation**	**Normal range**
Calcium (mM)	2.10	2.25–2.65
Phosphorous (mM)	2.30	0.90–1.70
PTH (pM)	1.2	1.2–5.5

His mother, cousin, grandmother, and great-grandmother were also affected with hypoparathyroidism revealing an autosomal dominant inheritance pattern. Neither the patient nor any of his family members had a history of mucocutaneous candidiasis, hearing loss, renal abnormalities, or skin changes. He was clinically diagnosed with ADH and genetic sequencing ruled out mutations in the *PTH* and *GCM2* genes, but also in *CASR*. Exome sequencing revealed a novel mutation in *GNA11*, which was present only in affected family members (Mannstadt et al., 2013).

Few patients have been described in the literature with ADH2, limiting the number of observations (Table 2), but ADH2 might have a slightly milder phenotype with respect to hypocalcemia than patients with ADH1. Additionally, elevations in urinary calcium of individuals with ADH2 appear to be less pronounced than in those with activating CASR mutations in ADH1, raising the question whether the CASR in the renal tubules couples to G proteins other than G11.

**Table 2:** **Summary of the biochemical characteristics of the families/patients described with ADH type 2**.

	**Family A (Mannstadt et al., 2013)**	**Family B (Mannstadt et al., 2013)**	**Patient 3 (Nesbit et al., 2013)**	**Patient 4 (Nesbit et al., 2013)**	**Li et al., 2014**	**Piret et al., 2016**	**Tenhola et al., 2016**
*GNA11* Mutation	Arg60Cys	Ser211Trp	Arg181Gln	Phe341Leu	Arg60Leu	Val340Met	Val340Met
No. of Individuals	6	8	1	1	3–4	1–4	7
Calcium (mmol/l) (nl range)	2.10±0.07 (2.15–2.70)	1.91±0.12 (2.10–2.55)	2.06 (2.1–2.5)	1.75 (2.1–2.5)	1.86^c^ (2.20–2.60)	1.88^d^ (2.20–2.60)	Adult-1.90^f^ (2.15–2.51) Child-2.03^h^ (2.05–2.70)
Phosphorus (mmol/l) (nl range)	1.92±0.76 (0.70–1.4)	1.79±0.37 (0.80–1.50)	1.09 (0.7–1.40)	1.54 (0.7–1.40)	2.16^c^ (0.8–1.8)	1.42–1.81^e^ (0.8–1.45)	Adult-1.21^g^ (0.71–1.53) Child-2.03^h^ (1.10–1.80)
PTH (nl range)	1.7±0.93 (1.0–7.0 pmol/l)	1.33±0.75 (1.6–6.9 pmol/l)	50 (10–65 ng/l)	1.3 (1.3–7.6 pmol/l)	1.3 (1.2–5.8 pmol/l)	7.78^d^ (12–65 pg/ml)	Adult-12^g^ (12–47 ng/l) Child 15^h^ (12–47 ng/l)
Magnesium (mmol/l) (nl range)	0.77^a^ (0.7–1.0)		0.77 (0.70–1.05)	0.76 (0.70–1.05)	0.83^c^ (0.7–1.0)		Adult-0.81^i^ (0.71–0.94) Child-0.82^h^ (0.70–1.0)
Urinary calcium (nl range)	Normal^b^ Ca:Cre Ratio	Normal^b^ Ca:Cre Ratio	Ca:Cre Clearance = 0.002 >0.02	Ca:Cre Clearance = 0.012 >0.02	FE_*Ca*_ = 0.91 (1.0–2.0)	24 h UCa = 4.3–10.2^e^ (2.5–7.5 mmol/24 h)	Adult 24 h UCa = 4.6^i^ (1.3–6.5 mmol/24 h) Child Ca:Cre Ratio = 0.15^h^ (< 0.6)

a Mean of 10 patients from both Family A and Family B.

b Measured in a total of 8 patients across both families.

c Mean taken from each individual's mean of 1–10 values.

d Average of 3–4 individuals, one of whom the median of a range is averaged.

e Range of one individual.

f One adult.

g Average of 3 adults.

h Average of 4 children.

i Average of 2 adults.

The genetic basis of ADH2 was first elucidated by two groups. One study focused on two large unrelated families counting a total of 15 living patients with autosomal dominant isolated hypoparathyroidism, in whom mutations in the genes encoding the CASR, PTH, and GCM2 were ruled out (Mannstadt et al., 2013). Affected individuals in both families were hypocalcemic with inappropriately low PTH and elevated phosphorous. The urinary calcium:creatinine ratio was found to be normal in all of 8 patients measured across both families. Genetic linkage analysis revealed a linked interval of ~10 Mb on chromosome 19p13.3 with a maximal LOD score of 3.0. Among the approximately 50 genes that reside at this locus, *GNA11* was a strong candidate gene. Sanger sequencing discovered a heterozygous missense mutation in *GNA11* exon 2 (c.178C->T) changing the conserved arginine 60 to a cysteine (p.Arg60Cys). Whole-exome sequencing of two members of this family revealed that this R60C was the only variant in the linked interval that was present in both subjects. In the second family, exome sequencing of two affected members uncovered a different mutation in *GNA11*, a heterozygous missense mutation in exon 5. This mutation (c.632C->G) leads to the substitution of the conserved serine 211 to a tryptophan (p.Ser211Trp). Analysis of exome data from both families confirmed the absence of other variants affecting the same gene in both families. Restriction site analysis of PCR products revealed the presence of the respective mutation in available affected members of each family, and the absence in unaffected members (Mannstadt et al., 2013).

Investigators of the other study discovered inactivating mutations in *GNA11* associated with the mirror-image disease FHH. They then hypothesized that activating mutations of *GNA11* could cause ADH (Nesbit et al., 2013). Eight unrelated patients with hypocalcemia and low or inappropriately normal PTH levels in the absence of *CASR* mutations were studied by Sanger sequencing. In two ADH patients, mutations in the *GNA11* gene were uncovered. These patients had normal magnesium levels, and normal urinary calcium to creatinine clearance. The mutations were heterozygous missense mutations in highly conserved residues, namely a G->A change at c.542 which predicts a protein change of Arg181Gln, and a C->G change at c.1023 which causes the Phe341Leu change (Nesbit et al., 2013).

Subsequently, additional mutations in *GNA11* in other families with ADH were published. In one large family, whole-exome-sequencing revealed a heterozygous mutation c.179G->T; p.R60L (Li et al., 2014). The affected individuals in this family were reported to have short stature relative to their unaffected family members. They also lacked hypercalciuria and had normal serum magnesium levels, which contrasts with patients with ADH1 (Li et al., 2014). A second large family from Iran with ADH was studied by whole-exome sequencing revealing a heterozygous mutation exon 7 of *GNA11* c1018G->A; p.Val340Met (Piret et al., 2016). The Val340Met mutation was also found in a family from Finland, in which all seven of the family members studied had short stature and three had intracranial calcifications (Tenhola et al., 2016).

*In vitro* studies of these *GNA11* mutants using HEK293 cells expressing the CASR have confirmed the hypothesis that they are gain-of-function mutations. A leftward shift in the intracellular calcium response curve to extracellular calcium was found, indicating increased intracellular response to lesser amounts of extracellular calcium in the setting of each mutant (Nesbit et al., 2013; Li et al., 2014; Piret et al., 2016). Likewise, cells expressing the R60L mutant demonstrated increased activation of the SRE promoter and increased phosphorylation of ERK and p38, downstream targets of G11/q signaling (Li et al., 2014). *In vitro* studies using HEK293 cells have shown that the calcilytic NPS2143, an allosteric modulator of the CASR, can rectify impaired calcium signaling in ADH2 despite the fact that the Gα11 protein is downstream of the CASR (Babinsky et al., 2016).

Molecular modeling has suggested different mechanisms through which these mutations confer a gain of function in Gα11 (Flock et al., 2015). The R60 residue (G.H1.9) is located in the GTPase domain and has polar interactions with Asp71 (H.HA.3), which stabilize the interaction of the GTPase and helical domain, thus stabilizing the GDP-bound inactive form of Gα11. Replacement of the R60 residue is predicted to loosen the tight clamshell leading to faster GDP-GTP exchange (Mannstadt et al., 2013). Substitution of Ser211 (G.H2.2) with the bulky tryptophan is predicted to disturb the interactions with the βγ subunits (Mannstadt et al., 2013). The Arg181 (H.HF.6) residue is located in the helical domain and forms hydrogen bonds with residue R183 that is important for GTPase activity and can confer oncogenic potential to G11 when mutated (Nesbit et al., 2013). The Val340Met (G.H5.7) and Phe341Leu (G.H5.8) mutations are located in the α5 helix of Gα11 therefore altering the contacts between H5 and H1, which are important for Gα subunit activation (Nesbit et al., 2013; Li et al., 2014).

To date, six mutations in GNA11 have been reported to cause ADH2 (Mannstadt et al., 2013; Nesbit et al., 2013; Li et al., 2014; Piret et al., 2016; Tenhola et al., 2016). The discovery of activating germline mutations in the ubiquitously expressed G11 protein was surprising, especially since apart from hypocalcemia, and small stature reported in two families, no other obvious phenotypes were found. Further, studies of these families and identification of new families, possibly with novel mutations, will increase our understanding of the action of Gα11 and refine the phenotypic characteristics of patients with ADH2.

## Conclusions and unanswered questions

In conclusion, much has been learnt from mutations in the CASR and its signaling molecule G11. Activating mutations of the CASR lead to ADH1 and activating mutations in Gα11 cause ADH2. Hypercalciuria is a particularly difficult problem in ADH1 due to two independent mechanisms: low PTH and activation of the CASR in the renal TAL. The use of small molecule negative CASR modulators (calcilytics) for treatment of ADH1 has the potential to correct defects both in the parathyroid glands and in the renal tubule. *In vitro* studies and experiments in mouse models of ADH1 have demonstrated the ability of calcilytics to stimulate PTH, increase serum calcium, and decrease urinary calcium. A preliminary report of a phase 2 clinical trial is consistent with the efficacy of calcilytics in ADH1 patients. Clearly, small molecule CASR inhibitors would be a very attractive treatment option and in line with the concept of “precision medicine” for patients with ADH1.

Many important questions about ADH1 and ADH2 remain and further research is needed. Do patients with germline activating mutations in Gα11 have other, perhaps more subtle phenotypes caused by the activation of Gα11 in other tissues? Do different mutations lead to different phenotypes? Do calcilytics alleviate the biochemical abnormalities *in vivo* in patients with Gα11 mutations? Is there a role for the recently described G11/q inhibitors YM254890 and FR900359 (Schrage et al., 2015; Xiong et al., 2016) as a treatment for ADH2? Could novel PTH agonists be useful in the treatment of ADH (Bi et al., 2015)? Investigating these questions could ultimately help define new drug targets and improve medical management of patients with ADH types 1 and 2.

## Author contributions

All authors listed, have made substantial, direct and intellectual contribution to the work, and approved it for publication.

### Conflict of interest statement

The authors declare that the research was conducted in the absence of any commercial or financial relationships that could be construed as a potential conflict of interest.
